# Assessment of parasite clearance following treatment of severe malaria with intravenous artesunate in Ugandan children enrolled in a randomized controlled clinical trial

**DOI:** 10.1186/s12936-018-2552-6

**Published:** 2018-10-30

**Authors:** Pauline Byakika-Kibwika, Patience Nyakato, Mohammed Lamorde, Agnes N. Kiragga

**Affiliations:** 1 Department of Medicine, College of Health Sciences, Makerere University, P. O. Box 7072, Kampala, Uganda; 2 Infectious Diseases Institute, Kampala, Uganda

**Keywords:** Artesunate, ACT, Malaria, Parasite clearance

## Abstract

**Background:**

Malaria control largely depends on availability of highly efficacious drugs, however, over the years, has been threatened by emergence of drug resistance. It is, therefore, important to monitor the impact of recurrent anti-malarial treatment on the long-term efficacy of anti-malarial regimens, especially in sub-Saharan African countries with high malaria transmission. Evaluation of parasite clearance following treatment of severe malaria with intravenous artesunate among patients in Eastern Uganda, was performed, as a contribution to monitoring anti-malarial effectiveness.

**Methods:**

Parasite clearance data obtained from a clinical trial whose objective was to evaluate the 42-day parasitological treatment outcomes and safety following treatment of severe malaria with intravenous artesunate plus artemisinin-based combination therapy among patients attending Tororo District Hospital in Eastern Uganda, were analysed. Serial blood smears were performed at 0, 1, 2, 4, 6, 8, 10, 12, 16, 20, 24 h, followed by 6-hourly blood smears post start of treatment until 6 h post the first negative blood smear when parasite clearance was achieved. Study endpoints were; parasite clearance half-life (the time required for parasitaemia to decrease by 50% based on the linear portion of the parasite clearance slope) and parasite clearance time (time required for complete clearance of initial parasitaemia).

**Results:**

One hundred and fifty participants with severe malaria were enrolled. All participants were treated with intravenous artesunate. All study participants tolerated artesunate well with rapid recovery from symptoms and ability to take oral mediation within 24 h. No immediate adverse events were recorded. The median (IQR) number of days to complete parasite clearance was of 2 (1–2). The median (IQR) time to clear 50% and 99% parasites was 4.8 (3.61–7.10) and 17.55 (14.66–20.66) h, respectively. The median estimated clearance rate constant per hour was 0.32. The median (IQR) slope half-life was 2.15 (1.64, 2.61) h.

**Conclusion:**

Parasite clearance following treatment with intravenous artesunate was rapid and adequate. This finding provides supportive evidence that resistance to artemisinins is unlikely to have emerged in this study area. Continuous monitoring of artemisinin effectiveness for malaria treatment should be established in high malaria transmission areas in sub-Saharan Africa where spread of resistance would be disastrous.

*Trial registration* The study was registered with the Pan African Clinical Trial Registry (PACTR201110000321348). Registered 7th October 2011, http://www.pactr.org/)

**Electronic supplementary material:**

The online version of this article (10.1186/s12936-018-2552-6) contains supplementary material, which is available to authorized users.

## Background

Worldwide, malaria ranks as one of the most important causes of morbidity and mortality with 216 million cases and 445,000 deaths in 2016 alone, 90% of cases and deaths occur in Africa and 80% in sub-Saharan Africa Uganda is one of the heavy malaria burden countries where *Plasmodium falciparum,* which causes the most severe form of disease is the most prevalent [1]. Malaria is transmitted by the female *Anopheles* mosquito which injects sporozoites into the human host. Sporozoites undergo pre-erythrocytic and erythrocytic stages of multiplication causing erythrocyte rupture with release of merozoites and pro-inflammatory cytokines into circulation, which are responsible for the symptoms. Patients seek medical attention when they begin to experience symptoms of malaria. Accurate diagnosis and prompt initiation of effective anti-malarial drugs is key to successful treatment of malaria, however, host immune mechanisms play a crucial role in parasite elimination [2–4].

In addition to vector control, malaria control largely depends on treatment with highly efficacious drugs, but over the years, this has been threatened by drug resistance. The artemisinin compounds currently recommended for malaria treatment are highly effective, however, there is significant risk for development of drug resistance if their use is not adequately controlled and monitored. Resistance to artemisinins which manifests as loss of the intracellular ring stage parasite susceptibility and slower parasite clearance [5], was reported initially on the Thai-Cambodia border [6, 7] and is likely to spread to other malaria endemic areas. It is imperative for malaria control programmes to establish mechanisms for monitoring artemisinin efficacy and risk for spread of artemisinin resistance especially in areas with high malaria transmission where recurrent malaria infections and treatment occur.

Monitoring of artemisinin efficacy is performed using parasite clearance curves constructed from sequential parasite densities following malaria treatment [8]. Previous studies demonstrated a lag phase in parasite clearance followed by a log-linear decline in parasitaemia following initiation of artemisinins. The slope of the log-linear phase of the parasite clearance curve or the derived half-life (time required for parasitaemia to decrease by 50%) can be used to predict resistance [5]. Artemisinin resistance is suspected when > 10% of patients treated with artemisinin-based combination therapy (ACT) or artesunate monotherapy have positive parasitaemia on day 3 or ≥ 10% of patients have parasites with a clearance half-life ≥ 5 h. Resistance to artemisinins is confirmed when parasites persist in the blood for 7 days or with presence of parasites at day 3 and recrudescence within 28 days after treatment with oral artemisinin based monotherapy. Presence of > 5% of patients carrying K13 resistance-confirmed mutations confirms artemisinin resistance [9–11]. Some authors indicate a more sensitive threshold of > 5% day 3 parasite positivity to trigger further investigation for artemisinin susceptibility [12]. This study aimed to evaluate malaria parasite clearance following treatment of patients with severe malaria using intravenous artesunate in Tororo District Hospital in Eastern Uganda.

## Methods

### Study design, site and population

Data obtained from a randomized clinical trial whose objective was to evaluate the 42-day parasitological treatment outcomes and safety following treatment of children with severe malaria using intravenous artesunate plus artemisinin combination therapy (artemether + lumefantrine or dihydroartemisinin + piperaquine) in Tororo District Hospital, in Eastern Uganda, were analysed [13]. Eastern Uganda has perennial malaria transmission and an annual entomological inoculation rate estimated to be 310 infective bites per person per year [14]. Consecutive patients presenting to the hospital with symptoms and signs of severe malaria and a positive thick blood film for malaria parasites were referred to the study physicians for further assessment for study eligibility. Patients were enrolled if they fulfilled the criteria for severe malaria requiring intravenous anti-malarial drugs. Patients were excluded if they had obvious concomitant febrile illness that would interfere with monitoring malaria treatment outcomes, history of allergy to any of the study drugs or if they had history of receipt of an effective anti-malarial drug within 24 h prior to presentation to hospital.

### Study procedures

Following diagnosis of severe malaria, participants were immediately initiated on intravenous artesunate (Guilin Pharmaceutical Factory, Guangxi, China) administered as 2.4 mg/kg at start of treatment, repeated at 12 h and every 24 h from start of treatment until the switch to oral therapy. Each 60 mg vial of artesunic acid was dissolved in 1 mL of 5% sodium bicarbonate and mixed with 5 mL of 5% dextrose, injected as a slow bolus into an indwelling IV cannula. Intravenous artesunate was administered for a minimum of 24 h and when participants could tolerate oral therapy, they were given a full course of either artemether + lumefantrine or dihydroartemisinin + piperaquine. All participants received paracetamol 15 mg/kg at 8 hourly intervals. Adjunctive and supportive treatment, such as diazepam for convulsions and dextrose for hypoglycaemia, was given in accordance with the Uganda Ministry of Health Malaria Treatment guidelines.

### Follow-up and laboratory procedures

Thin blood smears were performed to detect the malaria species at diagnosis. Thick blood smears were performed to calculate parasite density serially at 0 h (before start of treatment), 1, 2, 4, 6, 8, 10, 12, 16, 20, 24 h, followed by 6 hourly blood smears post start of treatment until 6 h post the first negative blood smear when parasite clearance was achieved. Thick blood smears were stained using 3% Giemsa for 30 min and read by experienced microscopists. All slides were read by two independent experienced microscopists and any discrepant results were reviewed by a third experienced microscopist. Parasite density was calculated by counting the number of asexual parasites per 200 white blood cells (WBCs) assuming a WBC count of 8000/μL of blood. Participants were discharged from hospital with a full course of either artemether + lumefantrine or dihydroartemisinin + piperaquine, when the blood smear was negative for malaria.

### Statistical analysis

Data were entered and verified using MS ACCESS and analysed using STATA version 13.1 (STATA Corporation, College Station, TX, USA). Descriptive statistics were summarized into medians and interquartile ranges (IQR). Parasite density was normalized using logarithmic transformation. Parasite clearance was estimated using the World Wide Anti-malarial Resistance Network (WWARN) parasite clearance estimator (PCE) algorithm [8, 12]. The algorithm involves a step-by-step process of obtaining the clearance rate constant for each individual patient based on the slope of the linear portion of the log-parasitaemia versus time curve. Data were cleaned to remove outliers or tails and to determine presence of lag phase in some of the parasite profiles. Parasite profiles that had less than three parasite profiles were not included in calculation of PCE. The detection limit was 8000 parasites per 200 WBCs equivalent to 40/μL. For any profile that had a zero last parasite count, this was replaced by the detection limit and a tobit regression fit. The curve of the log_e_ parasite count versus time was described mathematically using a polynomial model and lag phase identification done from the mathematical model. If a lag phase was not identified, the clearance rate constant was estimated as the absolute value of the slope of the linear regression model to all data. If a lag phase was identified, the clearance rate constant was then estimated as the absolute value of the slope from the linear part of the predicted profile.

### Ethical considerations

The study was approved by Makerere University School of Medicine Research and Ethics Committee (REC REF 2011-175), Uganda National Drug Authority (369/ESR/NDA/DID-12/2011), Uganda National Council for Science and Technology (HS 1031) and registered with the Pan African Clinical Trial Registry (PACTR201110000321348). All study procedures were conducted according to Good Clinical Practice standards. Patients and parents or guardians of participants provided written informed consent prior to enrollment. Study related information was provided in the participants’ local languages.

## Results

Data were obtained from 150 participants with severe malaria, treated with intravenous artesunate between December 2011 and April 2013. Baseline characteristics are shown in Table 1.

**Table 1:** Baseline characteristics of study participants

Characteristics	AS + DP N = 79	AS + AL N = 71
Female (%)	36 (45.6)	36 (50.7)
Age in months*	17 (11–26)	16 (10–26)
Weight (kg)*	9.5 (8–11.5)	9 (8.1–11)
Temperature (°C)*	38.8 (37.7–39.5)	39.1 (37.7–39.5)
Parasite density per μL, log10 copies*	4.82 (4.29–5.10)	4.79 (4.38–5.02)
Haemoglobin (mg/dL)*	9.1 (7.9–10.5)	9.2 (8.4–10.6)
Total white blood cell count (*10^3/^μL)*	9.6 (6.9–13.2)	9.2 (7.5–12.3)
Random blood sugar (mmol/L)*	7.3 (6.3–8.3)	6.8 (6.4–8.3)
Complications at admission, n (%)		
Patients with history of repeated convulsions n (%)	6 (7.6%)	3 (4.2%)
Patients with history of inability to feed	26 (33.0%)	24 (34.0%)
Patients with prostration (extreme weakness)	22 (27.9%)	15 (21.13%)
Haemoglobinuria	0	0
Jaundice	2 (2.5%)	0
Severe anaemia	0	0
Respiratory distress	2 (2.5%)	6 (8.5%)
Impaired consciousness	0	0
Abnormal bleeding	2 (2.5%)	1 (1.4%)
Hypoglycaemia	0	0

* Values are presented as medians (IQR)

All study participants tolerated artesunate very well, with rapid recovery from symptoms and ability to take oral medication within 24 h. No immediate serious adverse events were recorded. None of the participants required additional medication. The median (IQR) number of days to complete parasite clearance (PCT) was of 2 (1–2). A total of 1559 (42.5%) parasite density-time profiles were included in the parasite clearance analysis. The median estimated clearance rate constant per hour was 0.32. The median (IQR) clearance half-life was 2.15 (1.64, 2.61) h. None of the patients had clearance half-life greater than 5 h. None of the patients had parasitaemia on day 3. The time to clear 50%, 90%, 95% and 99% parasites is shown in Table 2.

**Table 2:** Summary of time to clear 50, 90, 95 and 99% of parasites

Statistic	Time median (IQR) hours
PCT50	4.80 (3.61, 7.10)
PCT90	10.20 (8.47, 12.05)
PCT95	12.48 (10.57, 14.75)
PCT99	17.55 (14.66, 20.60)

*PCT50* time to clear 50% of parasites, *PCT90* time to clear 90% of parasites, *PCT95* time to clear 95% of parasites, *PCT99* time to clear 99% of parasites

## Discussion

Parasite clearance following treatment of severe malaria using intravenous artesunate, among patients attending Tororo District Hospital in Eastern Uganda was evaluated. Time to clear parasites was significantly shorter compared to that proposed as indicator for artemisinin resistance. Artemisinin resistance is suspected to occur when > 10% of patients treated with ACT or artesunate monotherapy have positive parasitaemia on day 3 or a clearance half-life of ≥ 5 h [9–11]. These findings are consistent with reports from studies performed in some Asian countries including Bangladesh and Laos [15], as well as some African countries including Nigeria, Democratic Republic of Congo and Kenya which reported rapid parasite clearance times but differ from studies performed in Thailand and Cambodia where longer parasite clearance times and much higher proportions of patients with parasitaemia on day 3 were demonstrated [16]. Host factors including age, weight, partial immunity, disease severity, and route of drug administration in addition to parasite resistance, affect clearance kinetics in vivo. Elimination of malaria parasites within 24–48 h indicates full susceptibility of *P. falciparum* to artemisinin. It is possible that parasite clearance times were shorter than what has been documented from other studies because we evaluated parasite clearance following intravenous administration of artesunate, while most previous studies from Asia evaluated parasite clearance following oral administration of artemisinin treatment, however, the current findings are comparable to those from studies performed in the African countries in which oral artesunate was administered [7, 16].

The short parasite clearance times did not warrant assessment for resistance conferring mutations. Presence of *k13* resistance mutations in > 5% of patients confirms artemisinin resistance [9–11]. These data are consistent with initial reports from previous large studies that indicated rapid parasite clearance following treatment with intravenous artesunate [17] and provide additional evidence that resistance to artemisinins is unlikely to have spread to this study area.

Artesunate has been shown to clear malaria parasites fast from circulation and is currently the recommended drug of choice for treatment of severe malaria [6, 18]. Previous studies attributed its effectiveness to the favorable pharmacokinetics such as short time from administration to attainment of maximum concentration of 1–2 h and rapid plus extensive hydrolysis to dihydroartemisinin, the metabolite with more active anti-malarial properties [19–22]. The high initial plasma concentrations of artesunate and dihydroartemisinin are responsible for the rapid parasite clearance, however, since the drug is rapidly cleared from circulation, it should be followed with a full course of ACT to prevent recrudescence. Drug assays of artesunate were not evaluated in this study, but the parasite clearance data are quite consistent with these pharmacokinetics data.

Although parasite clearance is currently used as an indicator of anti-malarial drug effectiveness and resistance, some authors suggest serious limitations with its use since parasite clearance is dependent on a combination of host immune mechanisms, parasite biology and drug pharmacology. Host immunity is said to be the primary determinant of parasite clearance, suggesting that parasite clearance is an insensitive tool that may lead to overconfidence and underestimation of reduction in drug sensitivity especially in areas where host immunity to malaria may be well developed [23].

This study has some limitations that should be considered. Majority of study participants weighed less than 20 kg, however, all received 2.4 mg/kg of artesunate at start of treatment, repeated at 12 h and every 24 h from start of treatment until the switch to oral therapy. Currently this dose of artesunate is considered low for children weighing less than 20 kg, because of differences in pharmacokinetics, and lower exposure to the active metabolite dihydroartemisinin with lower body weight [24, 25], however, this study was performed before the current WHO recommendation of 3.0 mg/kg of artesunate for children weighing less than 20 kg. Nonetheless, under-dosing would be expected to prolong parasite clearance time, whereas the findings of this study were rapid parasite clearance, underscoring the conclusion that artemisinin resistance it is unlikely to have spread to study area.

Parasite density could have been overestimated by counting the number of asexual parasites per 200 WBCs assuming a WBC count of 8000/μL of blood because malaria parasitaemia based on assumed WBCs is generally higher than parasitaemia based on actual WBCs due to changes in the WBC count over time which could alter the ratio and introduce error in the estimation of parasite density [26].

## Conclusion

These findings contribute to the existing knowledge on parasite clearance properties of intravenous artesunate for treatment of severe malaria and provide evidence that artemisinin resistance is unlikely to have spread to this study area. Continuous monitoring of artemisinin effectiveness for malaria treatment should be established in high malaria transmission areas in sub-Saharan Africa where spread of resistance would be disastrous.

## Additional file

**Additional file 1.** Study Data set.
